# Ganglioside GD3 May Suppress the Functional Activities of Benign Skin T Cells in Cutaneous T-Cell Lymphoma

**DOI:** 10.3389/fimmu.2021.651048

**Published:** 2021-03-30

**Authors:** Miki Kume, Eiji Kiyohara, Yutaka Matsumura, Hanako Koguchi-Yoshioka, Atsushi Tanemura, Yuma Hanaoka, Mifue Taminato, Hiroki Tashima, Koichi Tomita, Tateki Kubo, Rei Watanabe, Manabu Fujimoto

**Affiliations:** ^1^ Department of Dermatology, Course of Integrated Medicine, Graduate School of Medicine, Osaka University, Osaka, Japan; ^2^ Department of Integrative Medicine for Allergic and Immunological Diseases, Course of Integrated Medicine, Graduate School of Medicine, Osaka University, Osaka, Japan; ^3^ Department of Plastic Surgery, Course of Organ Regulation Medicine, Graduate School of Medicine, Osaka University, Osaka, Japan

**Keywords:** cutaneous T-cell lymphoma, ganglioside, GD3, resident memory T cells, antitumor immunity

## Abstract

In cutaneous T-cell lymphoma (CTCL), which arises from skin-tropic memory T cells, malignant T cells and benign T cells are confined in the same skin lesions. It is thus difficult to evaluate the phenotypic characteristics and functional activities of benign T cells in CTCL. Disialoganglioside with three glycosyl groups (GD3) is increasingly expressed on the surface of solid malignant tumor cells and takes part in tumor progression and suppression of tumor immunity. However, the role of GD3 in CTCL is not well-understood. In this study, the malignant and benign T cells in CTCL skin lesions were distinguished by flow cytometry and their phenotypic characteristics were compared with those of T cells from control skin specimens. In CTCL skin lesions, the benign T cells included limited resident memory T cells (T_RM_), which are sessile in skin and known to exert strong antitumor function. The benign T cells showed diminished Th17 property, and the expression of GD3 was high in the malignant T cells. The expression of GD3 in the malignant T cells inversely correlated with IL-17A production from the benign CD4 T cells. GD3 from the malignant T cells was implied to be involved in suppressing the Th17 activity of the benign T cells independent of the regulation of T_RM_ differentiation in CTCL. Revealing the role of GD3 in inhibiting the production of IL-17A in CTCL would aid the understanding of the suppressive mechanism of the antitumor activity by malignant tumor cells.

## Introduction

Cutaneous T-cell lymphoma (CTCL) is a heterogeneous group of non-Hodgkin lymphoma, in which malignant T cells primarily develop in the skin (1). In the most major CTCL subtype (mycosis fungoides [MF]), the malignant cells represent resident memory T-cell (T_RM_) phenotype, which reside in the skin and do not recirculate, in the early stage (2). At the same time, CTCL skin lesions include the benign counterpart T cells, which are supposed to exert antitumor immunity. In various solid malignant tumors, the expression of T_RM_ markers, especially CD103, in tumor infiltrating T cells is associated with a stronger antitumor activity (3–5). From the perspective of pro-inflammatory cytokine production, skin lesions in early stage CTCL are dominated with Th1/Tc1 cells, with the production of IFNγ and IL-2, while the lesions in advanced stage CTCL are enriched with the expression of the Th2 cytokines IL-4, IL-5, and IL-10 (6). The Th2 cytokines from the malignant T cells in CTCL blood and skin lesions reportedly suppress the Th1 responses of the benign counterpart T cells (7). However, the phenotypic characteristics and functional activity of benign T cells in CTCL skin lesions is not well-understood, since both malignant cells and benign counterparts are the same T cells and are confined in the same skin lesions, which makes it difficult to separately analyzing these populations.

Gangliosides represent a family of acidic glycosphingolipids that are distributed on the surface of various cells (8). Although disialoganglioside with three glycosyl groups (GD3) is regarded as a minor ganglioside, it is expressed in most tissues and its expression is especially upregulated in tumor conditions, such as malignant melanoma, ovarian cancer, and leukemia (9–11). In these tumor conditions, GD3 has been reported to contribute not only to the promotion of tumor cells, but to the suppression of antitumor immunity by inhibiting the functional activities of dendritic cells and CD4 T cells (11–15). Regarding CTCL, overexpression of 9-O-acetylated form of GD3 on circulating CD4 T cells is associated with poor prognosis in Sézary syndrome (16). However, the contribution of lesional GD3 in pathogenesis of CTCL has not been examined.

Herein, we distinguished the malignant and benign T cells in CTCL skin lesions by flow cytometry and compared their phenotypic characteristics with those of T cells from control skin specimens.

## Materials and Methods

### Sample Collection

This study was conducted on human tissue samples. The study protocols were in accordance with the Declaration of Helsinki and were approved by the Institutional Review Board of ethical committee in Osaka University Hospital (approval number 20108 and 20158-2). Written informed consents were obtained from all study participants. Lesional skin specimens were obtained from 12 clinically and pathologically confirmed CTCL patients as summarized in **Table 1**: 11 mycosis fungoides (MF) cases and 1 lymphomatoid papulosis case. Immunohistochemical evaluation was also admitted by the pathologists. Among these 12 subjects, more than 100 malignant T cells were detected in the skin lesions from 10 subjects. Clinical information, such as disease stage, serum levels of thymus and activation-regulated chemokine (TARC), and soluble IL-2 receptor (sIL-2R), were obtained from each patient at the time of sample collection. Skin specimens from a total of 29 patients were also collected as surgical discards from the resection and reconstruction of breast cancer, head and neck carcinomas, or skin *in situ* malignant or benign tumors (average age: 53.3 years; 5 males and 24 females). These skin specimens, which were regarded as controls, were at least 3 cm apart from the malignant tumors.

**Table 1:** Patient information.

Age	Sex	Dx	Stage	Bx site	Lesion	Tx history other than topical corticosteroid (bold: within 6 months)
48	M	MF	IIA	Thigh	Plaque	UV, IFN, **ET**, **PSL**, **MTX**, BEX
71	F	MF	IIB	Back	Plaque	UV, **ET**, IFN, RT, **MTX**, **VP-16**, BEX
62	F	MF	IIB	Thigh	Tumor	**UV**, ET, **ACNU**, IFN, VP-16, BEX, RT, MTX, Moga, GEM, **BV**
51	M	LyP	T3N0M0	Back	Tumor	**UV**
84	F	MF	IVA2	Thigh	Tumor	UV, ET, IFN, **BEX**, **VP-16**, RT, **Moga**, **MTX**, **ACNU**
82	M	MF	IB	Back	Plaque	UV, **ET**, BEX, **IFN**, **Moga**
55	M	FMF	IB	Buttock	Patch	(-)
82	F	MF	IB	Knee	Patch	(-)
62	M	MF	IIB	Back	Tumor	(-)
74	M	MF	IIB	Hand	Tumor	RT, UV, **ET**, **VP-16**
69	F	MF	IB	Lower leg	Plaque	(-)
38	F	MF	IB	Thigh	Patch	(-)

FMF, folliculotropic MF; UV, PUVA/nbUVB/excimer; RT, radiation; IFN, IFNγ; ET, etretinate; ACNU, nimustine; GEM, gemcitabine; BEX, bexarotene; VP-16, etoposide; Moga, mogamulizumab; BV, brentuximab vedotin; PSL, oral prednisolone > 5 mg/day; MTX, methotrexiate.

### Isolation of Skin T Cells

After removal of subcutaneous fat, skin specimens were minced and digested for 2 hours with 3 mg/mL of collagenase type III in RPMI 1640 medium (Wako, Osaka, Japan). The isolated cells were washed and incubated overnight in Iscove’s modified medium (Wako) supplemented with 10% Fetal Bovine Serum, L-Alanyl-L-Glutamine, penicillin/streptomycin, and 3.5 μL/L β-mercaptoethanol before analysis. In **Figure 4**, cells were isolated by short-term explant culture in the presence of 100 IU/mL of IL-2 (Wako) and 20 ng/mL of IL-15 (Wako). A hundred thousand of the isolated cells were incubated with or without 10 μg/mL GD3 (Adipogen AG, Liestal, Switzerland) for 15 hours before flow cytometry analysis. Concentration of GD3 was determined according to previous reports on its plasma concentration, functional assays (10, 17, 18), and our titration results using control blood T cells (**Supplementary Figure S2C**)

### Flow Cytometry

Monoclonal antibodies and isotype controls were used for surface or intracellular staining with optimal concentration. The antibodies are listed in **Table 2**. Prior to the intracellular cytokine staining, cells were stimulated with phorbol 12-myristate 13-acetate (PMA, 50 ng/mL, Wako) and ionomycin (750 ng/mL, Wako) plus Golgi Plug (BD Biosciences, NJ, USA) for 4 to 5 hours. Cells were surface-stained, fixed, permeabilized, and stained for intracellular targets using BD Cytofix/Cytoperm (BD Biosciences) according to the manufacturer’s protocol. Dead cells were detected and excluded using LIVE/DEAD™ Fixable Dead Cell Stain Kit (Thermo Fisher Scientific, MA, USA). Analysis of the samples was carried out on FACSCanto II flow cytometer (BD Biosciences) and data were analyzed with Kaluza software (Beckman coulter, CA, USA). Gating strategies are shown in **Supplementary Figure S1**. In order to confirm the reproducibility of the data, the setting of the flow cytometer was kept identical and the frozen aliquot of T cells from the same donor was analyzed consistently for the detection of GD3.

**Table 2:** Antibodies for the flow cytometry.

Molecule	Clone	Company
CD3	SK7	Biolegend
CD4	OKT4	Biolegend
CD7	CD7-6B7	Biolegend
CD8a	RPA-T8	eBioscience
CD69	FN50	Biolegend
CD103	BerACT8	Biolegend
GD3	R24	Abcam
Mouse IgG3	polyclonal	Abcam
Siglec-7	6-434	Biolegend
IFNγ	4S.B3	Biolegend
IL-17A	BL168	Biolegend

### Statistical Analysis

Mann-Whitney U test or Wilcoxon signed rank test was applied to compare 2 groups using GraphPad Prism software (GraphPad Software, CA, USA). Kruskal-Wallis test, followed by Dunn’s multiple comparisons test, was performed for comparison among 3 groups. Spearman’s rank correlation coefficients were measured to analyze the correlation between 2 indexes. *P* < 0.05 was considered significant: *p* < 0.05 (*), *p* < 0.01 (**), and *p* < 0.001 (***). The averages, standard deviations (SD), and actual *p* values/*r* values are listed in **Supplementary Table S1**.

## Results

### Benign T Cells in CTCL Lesion Include Less CD69^+^CD103^+^ T_RM_

Given that both benign and malignant T cells exist in CTCL lesions, we first distinguished the benign and malignant T cells by flow cytometry (**Supplementary Figure S1**). We confirmed that large cells with higher front scatter are mostly CD4 T cells that lack the expression of CD7. Among the small T cells, CD4 T cells expressing CD7 were defined as benign CD4 T cells since we were not able to deny the possibility that the small CD4 T cells without CD7 expression also have the malignant potency. Although the control CD4 T cells included CD7-low population, CD7-negative population was not detected. Most CD8 T cells both from CTCL lesions and control skin specimens were found to express CD7.

**Figure 1:**
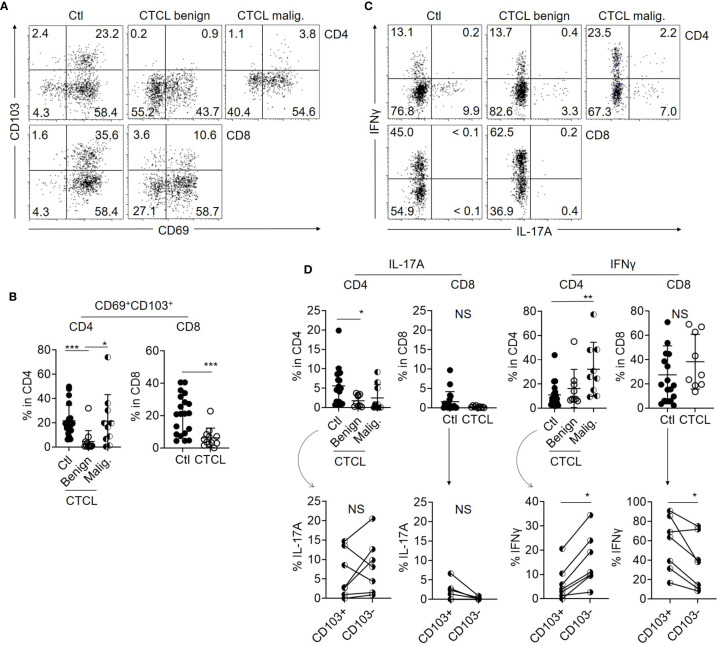
Benign T cells in CTCL lesions include less T_RM_ with limited Th17 property than those in control skin. **(A)** Representative dot plots showing the expression of CD69 and CD103 in CD4 and CD8 T cells from control skin specimens (Ctl), benign fraction, and malignant (malig.) fraction of CTCL lesions. **(B)** Graphs of the accumulated data showing the ratio of CD69^+^CD103^+^ cells in CD4 and CD8 T cells from Ctl, CTCL benign fraction, and malignant fraction. N = 20 in Ctl, n = 12 in CTCL benign, and n = 10 in CTCL malig. **(C)** Representative dot plots showing the production levels of IL-17A and IFNγ in CD4 and CD8 T cells from Ctl, CTCL benign fraction, and CTCL malignant fraction. **(D)** Top: Graphs of the accumulated data showing the production ratio of IL-17A and IFNγ in CD4 and CD8 T cells from Ctl, CTCL benign fraction, and CTCL malignant fraction. N = 14 in Ctl, n = 9 in CTCL. Bottom: In the Ctl CD4 and CD8 cells, the production ratio of IFNγ and IL-17A was compared between CD103^+^ and CD103^-^ fractions when the cytokines and CD103 were analyzed together. N = 7. *p < 0.05, **p < 0.01, ***p < 0.001. NS, not significant.

By comparing the expression levels of T_RM_ markers CD69 and CD103 between benign T cells in CTCL lesions and control skin T cells, it was found that the ratio of CD69^+^CD103^+^ T_RM_ was significantly lower in benign T cells from CTCL lesions than in control skin T cells both in CD4 and CD8 fractions (**Figures 1A, B**). CD103 expression was especially downregulated in CTCL benign T cells. The expression of T_RM_ markers was diverse in CTCL malignant T cells and this is in accordance with a previous report (19). These data demonstrate that benign T cells in CTCL lesions show diminished T_RM_ phenotype. The correlation between T_RM_ phenotype and disease stage was not observed (**Supplementary Figure S2B**).

### IL-17A Production Is Impaired in Benign CD4 T Cells From CTCL Lesions

In order to compare the cellular activity, the production levels of IL-17A and IFNγ were compared between CTCL lesional T cells and control skin T cells after stimulation with PMA and ionomycin. While IFNγ production was high in the malignant T cells and comparable between CTCL benign T cells and control skin T cells (**Figures 1C, D**), the production of IL-17A was significantly reduced in CTCL benign CD4 T cells when compared to control skin CD4 T cells (**Figures 1C, D**). The production of IL-17A from CD8 T cells was low both in CTCL lesions and control skin specimens (**Figures 1C, D**). These results suggest that production of the pro-inflammatory cytokine IL-17A is impaired in CTCL benign CD4 T cells, which are supposed to exert antitumor immunity. In terms of T_RM_ phenotype, the production of IL-17A was comparable between CD103^+^ and CD103^-^ populations in CD4 fraction (**Figure 1D**). The production level of IFNγ was higher in CD103^-^ population in CD4 T cells. Thus, the relation of T_RM_ marker expression and cytokine production was not proved in our analysis of CD4 T cells, although T_RM_ are generally considered to be associated with stronger effector function. On the other hand, the production of these cytokines from CD8 fraction tended to be dominated from CD103^+^ fraction (**Figure 1D**).

### Malignant T Cells in CTCL Skin Lesions Highly Express Ganglioside GD3

**Figure 2:**
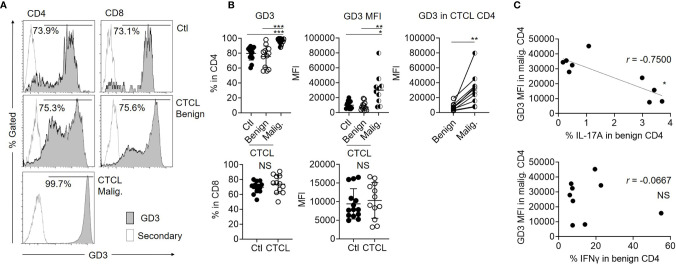
Malignant T cells from CTCL lesions express higher ganglioside GD3 compared to their benign counterparts and controls. **(A)** Representative histograms for GD3 expression of Ctl T cells, benign, and malignant T cells from CTCL lesions. Empty histograms indicate the controls stained by secondary antibody. **(B)** Left: Graphs of the accumulated data showing the ratio of GD3 expressing cells in CD4 (top) and CD8 (bottom) fractions of Ctl, CTCL benign fraction, and malignant fraction. N = 14 in Ctl, n = 12 in CTCL benign, n = 10 in CTCL malig. Data on CTCL malignant were excluded when the analyzed cell number was below 100. Middle: MFI of GD3 in CD4 (top) and CD8 (bottom) fractions of Ctl, CTCL benign fraction, and malignant fraction. N = 14 in Ctl, n = 12 in CTCL benign, n = 10 in CTCL malig. Right; MFI of GD3 compared between the benign and malignant CD4 T cells from the same CTCL lesions. N = 10. **(C)** Correlation between the MFI of GD3 in CTCL malignant T cells and the production ratio of IL-17A (top) and IFNγ (bottom) from CTCL benign CD4 T cells. N = 9. *p < 0.05, **p < 0.01, ***p < 0.001. NS, not significant.

Considering the possibility that the IL-17A-producing activity of benign CD4 T cells is suppressed by the malignant T cells in CTCL lesions, we next focused on ganglioside GD3, which is highly expressed in malignant cells of solid cancers. When the expression of GD3 was investigated in CTCL lesional T cells and control skin T cells, the ratio of GD3-expressing T cells was significantly higher in malignant T cells from CTCL lesions compared to their benign counterparts and control skin T cells (**Figures 2A, B**). Among the same CTCL skin lesions, the expression intensity (mean fluorescence intensity: MFI) of GD3 was significantly higher in the malignant T cells than in the benign counterpart CD4 T cells (**Figures 2A, B**). The expression of GD3 in CD8 fraction was comparable between CTCL and control skin specimens (**Figures 2A, B**). On the basis of these results, malignant T cells in CTCL skin lesions was found to express a high level of GD3, which is similar to that of malignant cells in solid tumors. The expression level of GD3 in the malignant T cells was inversely correlated with the production of IL-17A in the benign CD4 T cells, thus implying the involvement of GD3 in regulation of Th17 in the CTCL lesion (**Figure 2C**). Correlation was not found between IFNγ and GD3 (**Figure 2C**).

In terms of clinical correlation, neither the expression of GD3 in the malignant T cells nor the production of IL-17A in the benign CD4 T cells was reflected by the disease stage (**Supplementary Figure S2A**). The serum TARC level and sIL-2R level did not show any significant correlation with the MFI of GD3 in the malignant T cells or the IL-17A production from the benign CD4 T cells (**Supplementary Figure S2A**), although these indexes are known to indicate the disease activity in CTCL (20, 21).

### Benign CD4 T Cells From CTCL Skin Lesions Contain Siglec-7-Expressing Cells

**Figure 3:**
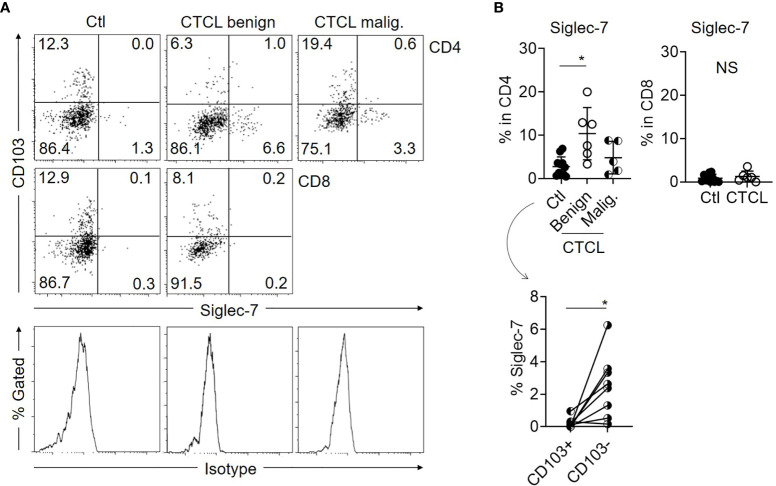
Expression of siglec-7 is augmented in benign CD103^-^ CD4 T cells of CTCL lesions. **(A)** Representative dot plots showing the expression of CD103 and siglec-7 in Ctl T cells, benign and malignant T cells from CTCL lesions. Histograms indicate the isotype controls. **(B)** Top: Graphs of the accumulated data showing the ratio of siglec-7-expressing cells in CD4 (left) and CD8 (right) fractions of Ctl, CTCL benign fraction, and malignant fraction. N = 11 in Ctl, n = 6 in CTCL benign, n = 5 in CTCL malig. Bottom: The ratio of siglec-7^+^ cells in CD103^+^CD4 and CD103^-^CD4 fractions of Ctl skin specimens. N = 8. *p < 0.05. NS, not significant.

Siglecs are the known ligands for GD3 and are involved in suppressive signal transduction *via* the recruitment of SH-2-containing phosphatase 1 (SHP-1) (22–24). Among the siglecs, siglec-7 and siglec-9 are expressed on NK cells, T cells, and neutrophils and are generally reported to suppress antitumor immunity (23, 25). Herein, we focused on siclec-7, since a contradictory function has been reported about its suppressive role (22, 25, 26). When the expression level of siglec-7 was examined in T cells from CTCL lesional skin and control skin specimens, it was found that a part of the benign CD4 T cells in CTCL lesions express siglec-7, while the expression of siglec-7 on the malignant T cells, benign CD8 T cells in CTCL, and control skin T cells was limited (**Figures 3A, B**). The expression of siglec-7 was mainly observed in CD103^-^ CD4 T-cell fraction, which includes non-T_RM_ (**Figure 3B**).

### GD3 Partially Suppresses the Production of IL-17A From Skin CD4 T Cells

**Figure 4:**
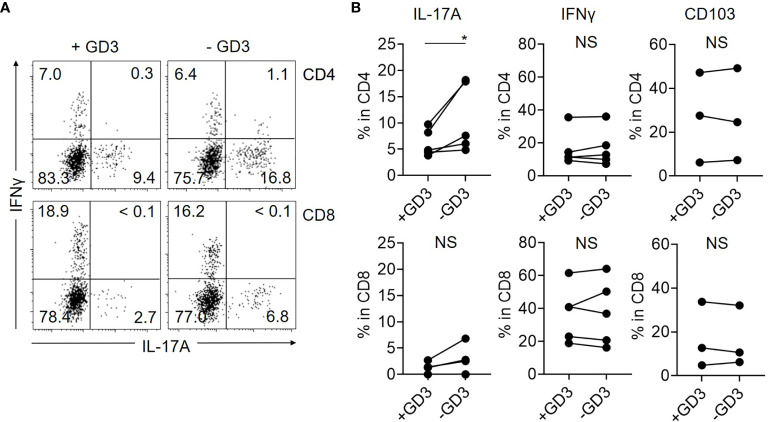
GD3 suppresses the production of IL-17A by CD4 T cells. T cells from Ctl skin specimens were incubated with or without 10 µg/mL of GD3 and the cytokine production was assessed by flow cytometry. **(A)** Representative dot plots showing the production of IL-17A and IFNγ from CD4 (top) and CD8 (bottom) fractions of Ctl skin specimens after incubation with (left) or without (right) GD3. **(B)** Graphs of the accumulated data showing the producing ratio of IL-17A (left) and IFNγ (middle) in CD4 (top) and CD8 (bottom) fractions. N = 5. Right: Expression level of CD103 after incubating CD4 (top) and CD8 (bottom) T cells from Ctl skin specimens with or without GD3. N = 3. *p < 0.05. NS, not significant.

Based on the hypothesis that GD3 from malignant T cells suppresses the cytokine-producing activities of benign T cells in CTCL lesions, we cultured T cells isolated from control skin specimens with or without GD3. Then, IL-17A production was significantly reduced from CD4 T cells in the presence of GD3, although the suppression was partial (**Figures 4A, B**). The production level of IFNγ was not obviously affected by GD3 and, regardless of GD3, the expression of CD103 was also comparable (**Figure 4B**). Thus, the augmented GD3 in CTCL lesions is considered to, at least, partially suppress IL-17A production from the benign CD4 T cells, possibly *via* siglec-7, which is dominantly expressed in CD103 fraction. The correlation between the expression of siglec-7 and GD3 was not successfully assessed due to the sample limitation.

## Discussion

Herein, we demonstrate that benign T cells in CTCL skin lesions consist of less T_RM_ compared to control skin specimens. The benign CD4 T cells in CTCL lesions are inferior to the control skin CD4 T cells in their ability to produce IL-17A. This diminished IL-17A production is implied to be, at least in part, due to the augmented GD3 production from the malignant T cells. Actually, a subpopulation of the benign CD4 T cells in CTCL lesions express siglec-7, which is one of the inhibitory GD3 ligands. It is implied that benign T_RM_ differentiation in CTCL lesion and production of IL-17A is regulated independently and GD3 mainly affects IL-17A production from the benign T cells.

Recent researches have revealed the distinct roles of CD4 and CD8 T_RM_ in their tissue protection against pathogens (27–32). In solid malignant tumor settings, the infiltration of T cells expressing T_RM_ markers correlates with strong antitumor function and good prognosis, especially in CD8 fraction (5, 33–36). On the other hand, the antitumor role of CD4 T_RM_ has not been well-elucidated (37). In our study, both CD4 and CD8 benign T_RM_ were reduced in CTCL lesion. However, the trend of correlation between pro-inflammatory cytokines and the T_RM_ marker was found only in CD8 fraction, although T_RM_ phenotype, especially CD103 expression, is reportedly linked to the production of IL-17A (38–40). T_RM_ phenotype and IL-17A production in benign T cells was comparable between the early stage and advanced stage. It is possible that our results are skewed by the limited sample number and the treatments modification. Therefore, further accumulation of data will be needed.

Our results demonstrate the downregulated Th17 activity of the benign T cells in CTCL lesions. Depending on the tumor types and stages, Th17 reportedly has a dual function of promoting both cancer progression and antitumor immunity (41–44). In the context of lymphoma, the production of IL-17 correlates with the tumor burden in classical Hodgkin lymphoma, thus suggesting the involvement of IL-17 from tumor cells in tumor progression (45). On the other hand, in an intraocular lymphoma model, IL-17 expression in infiltrating CD4 T cells inversely correlated with tumor burden (46). The involvement of IL-17A in CTCL pathogenesis is also controversial. According to a previous report, skin T cells from leukemic CTCL showed a limited production of IL-17A both before and after successful treatments (2). Moreover, a relatively low expression of IL-17A was also mentioned in the lesional skin of CTCL (47). Although other reports demonstrate the upregulated production of IL-17A/F in CTCL specimens and cell lines (48–50), the autocrine mechanism of IL-17A was not shown in the malignant cells. The functional deviation of Th17 cells is also suggested to be accompanied with the impaired production of antimicrobial peptides in CTCL lesions (51). These reports imply that IL-17A may not strongly take part in the tumor progression, but may function in antitumor immunity in CTCL.

GD3 reportedly promotes the malignant properties of cancer cells (11, 52–54) and suppresses antitumor activities (10, 22). Although the benign and malignant cells were both the same T cells in CTCL, the expression level of GD3 was distinct with a significantly high expression in the malignant T cells, which is similar with those of other tumors. In addition, the expression level of GD3 in the malignant cells inversely correlated with the production level of IL-17A in the benign CD4 T cells from CTCL lesions. Although they not only inhibit antitumor immunity (13–15), gangliosides are known to suppress inflammatory reactivity in murine multiple sclerosis model, a systemic Th17 disease condition (55). In addition, siglecs, which serve as ligands of GD3, contain immunoreceptor tyrosine-based inhibitory motif in their cytoplasmic domains and recruit Src homology-2 domain-containing protein-tyrosine phosphatase-1 (SHP-1) (56). SHP-1 reportedly suppresses Th17 reactivity by decreasing the phosphorylation of STAT3 in CD4 T cells (57). Although we were not able to prove this mechanistic pathway in this study, our results imply that GD3 may regulate IL-17A production in benign CD4 T cells of CTCL by activating SHP-1 and suppressing the phosphorylation of STAT3 *via* siglec-7.

This study includes the following limitations. The number of the samples is small and we were not able to confirm the relation of lesional GD3 expression and the disease severity/activity. Also, the correlation between IL-17A production and disease severity/activity was not confirmed. We admit that our analysis, especially the cellular GD3 expression and the clinical indexes, were possibly affected by the treatments. The working mechanisms of GD3 suppression of IL-17A production was not investigated in detail in this study. Among the siglecs, although siglec-7 and siglec-9 are reportedly involved in the same signaling pathway, we were not able to evaluate the expression of siglec-9 due to sample limitation. How the impaired IL-17A production and T_RM_ differentiation is involved in the pathogenesis of CTCL was not revealed. These limitations should be overcome in the near future.

Consequently, our results suggest that malignant T cells suppress Th17 activity of their benign counterpart T cells in CTCL lesions possibly *via* GD3 signaling, independent of the regulation of T_RM_ differentiation in CTCL. Our results suggest GD3 as a potential treatment target in CTCL.

## Data Availability Statement

The original contributions presented in the study are included in the article/**Supplementary Material**. Further inquiries can be directed to the corresponding author.

## Ethics Statement

The studies involving human participants were reviewed and approved by The Institutional Review Board of ethical committee in Osaka University Hospital. The patients/participants provided their written informed consent to participate in this study.

## Author Contributions

Conceptualization – MK, EK, RW, and MF. Data curation – MK, EK, YM, HK-Y, AT, YH, MT, HT, KT, TK, and RW. Formal analysis – MK, EK, and RW. Funding acquisition – RW and MF. Investigation – MK, EK, and RW. Methodology – MK, EK, and RW. Project administration – MK, EK, RW, and MF. Resources – All authors. Supervision – RW and MF. Validation – all authors. Visualization – MK, EK, and RW. Writing – original draft – MK, EK, and RW. Writing – review and editing – all authors. All authors contributed to the article and approved the submitted version.

## Funding

This research is supported by Grant-in-Aid for Scientific Research (KAKENHI) 16K19705 (to RW).

## Conflict of Interest

The authors declare that the research was conducted in the absence of any commercial or financial relationships that could be construed as a potential conflict of interest.

## Supplementary Material

Supplementary Figure 1 Gating strategy. The large cells and small cells were distinguished from forward scatter (FCS) and side scatter (SSC) dot plot panel and dead cells were excluded. Malignant cells were defined from the live large cells as CD4 positive and CD7 negative population. Benign CD4 T cells were defined from the live small cells as CD4 positive and CD7 positive. The small CD8 T cells were over 95% positive for CD7 and were all regarded as benign CD8 T cells. From Ctl skin, only one population was detected from the FCS and SSC dot plot panel. The empty histogram indicates the isotype control for CD7.

Supplementary Figure 2 Correlation of GD3 and IL-17A with clinical indexes. **(A)** Left: GD3 MFI in malignant CD4 T cells (top) and the ratio of IL-17A-producing cells in benign CD4 T cells (bottom) were compared between the CTCL patients with stage IB or lower (– IB, n = 5) and those with stage IIA or higher (IIA –, n = 5 in GD3, n = 4 in IL-17A). Middle and right: Correlation of serum TARC level (middle) and sIL-2R level (right) with GD3 MFI in malignant CD4 T cells (top) and the ratio of IL-17A-producing cells in benign CD4 T cells (bottom). **(B)** Ratio of CD69^+^CD103^+^ T_RM_ in the benign CD4 (left) and CD8 (right) T cells were compared between the CTCL patients with – IB (n = 5) and those with IIA – (n = 5). **(C)** Representative graph showing the ratio of IFNγ-producing cells in blood CD4 (white) and CD8 (gray) T cells after culture with the indicated concentrations of GD3 for 15 hours.

Supplementary Table 1 Statistical results. The numbers of subjects, averages, SD, statistical methods, and *p* values/*r* values in each figure are listed.
