# Supplementary figures and images for: Ganglioside GD3 May Suppress the Functional Activities of Benign Skin T Cells in Cutaneous T-Cell Lymphoma
Source: Front Immunol. 2021 Mar 30;12:651048. doi: 10.3389/fimmu.2021.651048 (PMC8042233; doi:10.3389/fimmu.2021.651048)

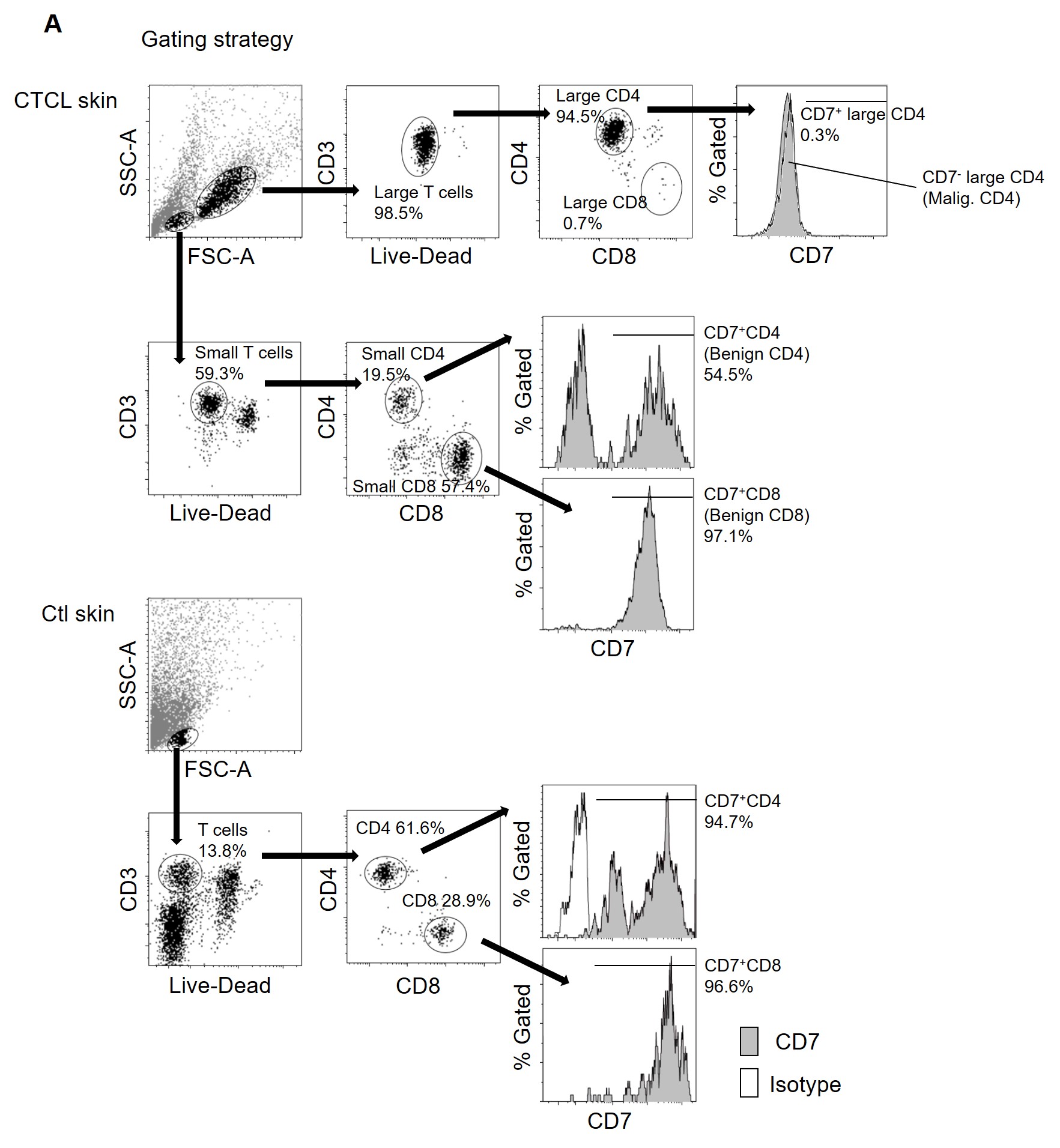

Supplement: Supplementary Figure 1 — Gating strategy. The large cells and small cells were distinguished from forward scatter (FCS) and side scatter (SSC) dot plot panel and dead cells were excluded. Malignant cells were defined from the live large cells as CD4 positive and CD7 negative population. Benign CD4 T cells were defined from the live small cells as CD4 positive and CD7 positive. The small CD8 T cells were over 95% positive for CD7 and were all regarded as benign CD8 T cells. From Ctl skin, only one population was detected from the FCS and SSC dot plot panel. The empty histogram indicates the isotype control for CD7. [file Image_1.jpeg]

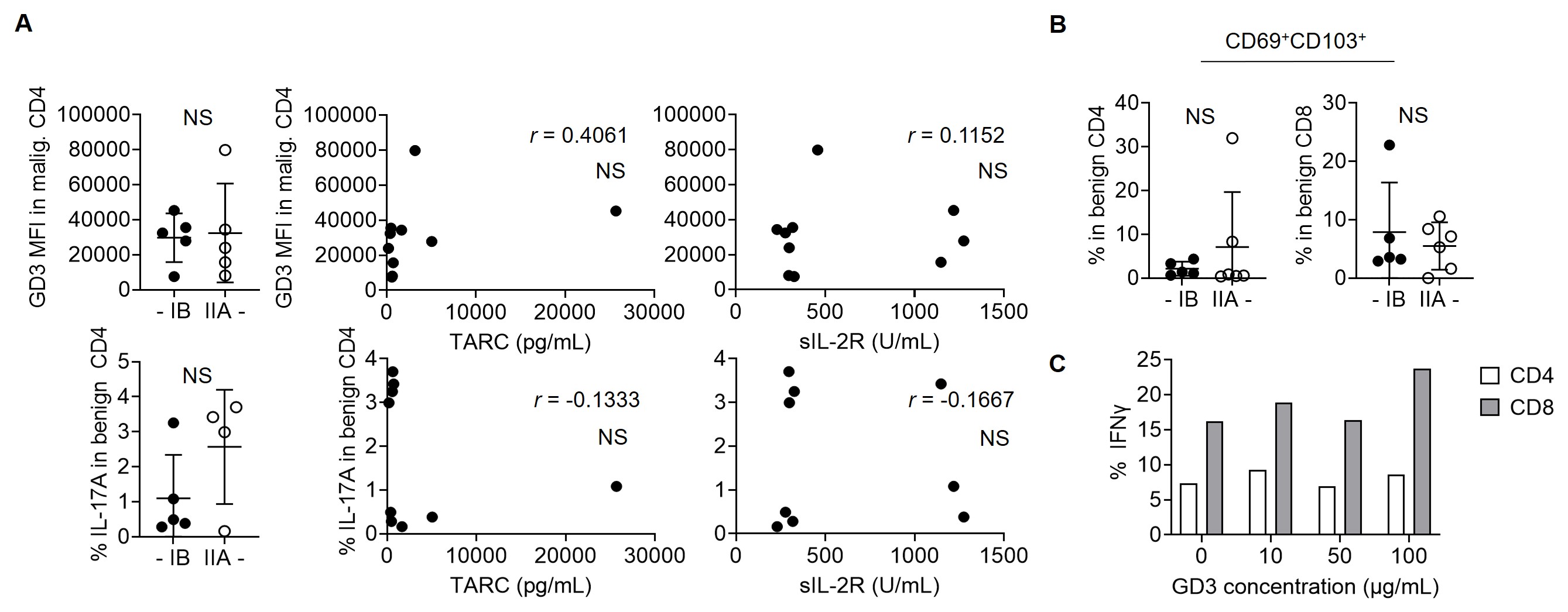

Supplement: Supplementary Figure 2 — Correlation of GD3 and IL-17A with clinical indexes. (A) Left: GD3 MFI in malignant CD4 T cells (top) and the ratio of IL-17A-producing cells in benign CD4 T cells (bottom) were compared between the CTCL patients with stage IB or lower (– IB, n = 5) and those with stage IIA or higher (IIA –, n = 5 in GD3, n = 4 in IL-17A). Middle and right: Correlation of serum TARC level (middle) and sIL-2R level (right) with GD3 MFI in malignant CD4 T cells (top) and the ratio of IL-17A-producing cells in benign CD4 T cells (bottom). (B) Ratio of CD69+CD103+ TRM in the benign CD4 (left) and CD8 (right) T cells were compared between the CTCL patients with – IB (n = 5) and those with IIA – (n = 5). (C) Representative graph showing the ratio of IFNγ-producing cells in blood CD4 (white) and CD8 (gray) T cells after culture with the indicated concentrations of GD3 for 15 hours. [file Image_2.jpeg]
