# Supplementary material for: Ganglioside GD3 May Suppress the Functional Activities of Benign Skin T Cells in Cutaneous T-Cell Lymphoma
Source: Front Immunol. 2021 Mar 30;12:651048. doi: 10.3389/fimmu.2021.651048 (PMC8042233; doi:10.3389/fimmu.2021.651048)
Supplement: Supplementary Table 1 — Statistical results. The numbers of subjects, averages, SD, statistical methods, and p values/r values in each figure are listed. [file DataSheet_1.pdf]

Figure		Average ± SD	Statistical method and p value/r value	Compared groups
1B	**% CD69+CD103+ in CD4**		**Kruskal-Wallis test/Dunn's multiple comparison test**	
	Ctl (n = 20)	21.73 ± 12.74	*** $p = 0.0003$	Ctl vs Benign
	Benign (n = 12)	4.54 ± 8.94	$p > 0.9999$	Ctl vs Malig.
	Malig. (n = 10)	21.28 ± 21.88	* $p = 0.0245$	Bening vs Malig.
	**% CD69+CD103+ in CD8**		**Mann-Whitney test**	
	Ctl (n = 20)	20.55 ± 12.17	*** $p = 0.0003$	Ctl vs CTCL
	CTCL (n = 12)	6.24 ± 6.04		
1D	**% IFNγ in CD4**		**Kruskal-Wallis test/Dunn's multiple comparison test**	
	Ctl (n = 18)	11.06 ±10.28	$p > 0.9999$	Ctl vs Benign
	Benign (n = 9)	16.27 ± 15.75	** $p = 0.0039$	Ctl vs Malig.
	Malig. (n = 9)	32.13 ± 22.28	$p = 0.1394$	Bening vs Malig.
	**% IFNγ in CD4 (Paired Ctl)**		**Wilcoxon signed-rank test**	
	% IFNγ in CD103+CD4 (n = 7)	6.45 ± 7.07	* $p = 0.0156$	CD103+ vs CD103-
	% IFNγ in CD103-CD4 (n = 7)	15.74 ± 10.77		
	**% IFNγ in CD8**		**Mann-Whitney test**	
	Ctl (n = 18)	27.41 ± 23.90	$p = 0.1936$	Ctl vs CTCL
	CTCL (n = 9)	38.22 ± 22.62		
	**% IFNγ in CD8 (Paired Ctl)**		**Wilcoxon signed-rank test**	
	% IFNγ in CD103+CD8 (n = 7)	56.54 ± 28.11	* $p = 0.0313$	CD103+ vs CD103-
	% IFNγ in CD103-CD8 (n = 7)	36.98 ± 27.91		
	**% IL-17A in CD4**		**Kruskal-Wallis test/Dunn's multiple comparison test**	
	Ctl (n = 18)	5.59 ± 4.78	* $p = 0.0455$	Ctl vs Benign
	Benign (n = 9)	1.75 ± 1.54	$p = 0.0524$	Ctl vs Malig.
	Malig. (n = 9)	2.49 ± 3.49	$p > 0.9999$	Bening vs Malig.
	**% IL-17A in CD4 (Paired Ctl)**		**Wilcoxon signed-rank test**	
	% IL-17A in CD103+CD4 (n = 7)	6.20 ± 6.08	$p = 0.2969$	CD103+ vs CD103-
	% IL-17A in CD103-CD4 (n = 7)	8.27 ± 6.91		
	**% IL-17A in CD8**		**Mann-Whitney test**	
	Ctl (n = 18)	1.57 ± 2.58	$p = 0.268$	Ctl vs CTCL
	CTCL (n = 9)	0.22 ± 0.23		
	**% IL-17A in CD8 (Paired Ctl)**		**Wilcoxon signed-rank test**	
	% IL-17A in CD103+CD8 (n = 7)	1.88 ± 2.41	$p = 0.1250$	CD103+ vs CD103-
	% IL-17A in CD103-CD8 (n = 7)	0.21 ± 0.33		
2B	**% GD3 in CD4**		**Kruskal-Wallis test/Dunn's multiple comparison test**	
	Ctl (n = 14)	79.80 ± 9.00	$p > 0.9999$	Ctl vs Benign
	Benign (n = 12)	75.38 ± 14.41	*** $p = 0.0008$	Ctl vs Malig.
	Malig. (n = 10)	96.25 ± 3.86	*** $p = 0.0002$	Benign vs Malig.
	**GD3 MFI in CD4**		**Kruskal-Wallis test/Dunn's multiple comparison test**	
	Ctl (n = 14)	10628 ± 4957	$p > 0.9999$	Ctl vs Benign
	Benign (n = 12)	8726 ± 5484	* $p = 0.0186$	Ctl vs Malig.
	Malig. (n = 10)	31069 ± 21031	** $p = 0.0018$	Benign vs Malig.
	**GD3 MFI in CTCL CD4 (Paired)**		**Wilcoxon signed-rank test**	
	Benign (n =10)	7414 ± 4868	** $p = 0.0020$	Benign vs Malig.
	Malig. (n = 10)	31069 ± 21031		
	**% GD3 in CD8**		**Mann-Whitney test**	
	Ctl (n = 14)	69.62 ± 7.48	$p = 0.3217$	Ctl vs CTCL
	CTCL (n = 12)	73.76 ± 12.02		
	**GD3 MFI in CD8**		**Mann-Whitney test**	
	Ctl (n =14)	9389 ± 4072	$p = 0.5604$	Ctl vs CTCL
	CTCL (n =12)	10316 ± 4789		
2C	**GD3 MFI in malig. CD4 vs % IL-17A in benign CD4**		**Spearman's rank correlation coefficient**	
	CTCL (n =9)		* $p = 0.0255$/$r = -0.7500$	
	**GD3 MFI in malig. CD4 vs % IFNγ in benign CD4**		**Spearman's rank correlation coefficient**	
	CTCL (n = 9)		$p = 0.8801$/$r = -0.0667$	
3B	**% siglec-7 in CD4**		**Kruskal-Wallis test/Dunn's multiple comparison test**	
	Ctl (n = 11)	2.77 ± 2.23	* $p = 0.0156$	Ctl vs Benign
	Benign (n = 6)	10.36 ± 6.04	$p = 0.7062$	Ctl vs Malig.
	Malig. (n = 5)	4.82 ± 3.73	$p = 0.5967$	Benign vs Malig.
	**% siglec-7 in CD4 (Paired Ctl)**		**Wilcoxon signed-rank test**	

	% siglec-7 in CD103+CD4 (n = 8)	0.21 ± 0.33	* *p* = 0.0156	CD103+ vs CD103-
	% siglec-7 in CD103-CD4 (n = 8)	2.52 ± 1.95		
	**% siglec-7 in CD8**		**Mann-Whitney test**	
	Ctl (n = 11)	0.87 ± 0.92	*p* = 0.6775	Ctl vs CTCL
	CTCL (n = 6)	1.26 ± 1.25		
**4B**	**% IL-17A in CD4**		**Wilcoxon signed-rank test**	
	+GD3 (n = 5)	6.21 ± 2.60	* *p* = 0.0313	+GD3 vs -GD3
	-GD3 (n = 5)	10.92 ± 6.57		
	**% IFNγ in CD4**		**Wilcoxon signed-rank test**	
	+GD3 (n = 5)	15.98 ± 11.22	*p* = 0.3125	+GD3 vs -GD3
	-GD3 (n = 5)	16.98 ± 11.41		
	**% CD103 in CD4**		**Wilcoxon signed-rank test**	
	+GD3 (n = 3)	26.99 ±20.52	*p* > 0.9999	+GD3 vs -GD3
	-GD3 (n = 3)	26.99 ± 21.10		
	**% IL-17A in CD8**		**Wilcoxon signed-rank test**	
	+GD3 (n = 5)	1.08 ± 1.13	*p* = 0.2500	+GD3 vs -GD3
	-GD3 (n = 5)	2.43 ± 2.79		
	**% IFNγ in CD8**		**Wilcoxon signed-rank test**	
	+GD3 (n = 5)	37.06 ± 17.02	*p* > 0.9999	+GD3 vs -GD3
	-GD3 (n = 5)	37.62 ± 20.05		
	**% CD103 in CD8**		**Wilcoxon signed-rank test**	
	+GD3 (n = 3)	17.10 ± 14.99	*p* = 0.5000	+GD3 vs -GD3
	-GD3 (n = 3)	16.33 ± 13.86		
**S2A**	**GD3 MFI in malig.CD4 vs TARC**		**Spearman's rank correlation coefficient**	
	CTCL (n = 10)		*p* = 0.2475/*r* = 0.4061	
	**GD3 MFI in malig.CD4 vs sIL-2R**		**Spearman's rank correlation coefficient**	
	CTCL (n = 10)		*p* = 0.7589/*r* = 0.1152	
	**% IL-17A in benign CD4 vs TARC**		**Spearman's rank correlation coefficient**	
	CTCL (n = 9)		*p* = 0.7435/*r* = -0.1333	
	**% IL-17A in benign CD4 vs sIL-2R**		**Spearman's rank correlation coefficient**	
	CTCL (n = 9)		*p* = 0.6777/*r* = 0.1667	
	**GD3 MFI in malig. CD4 vs stage**		**Mann-Whitney test**	
	- Stage IB (n = 5)	29733 ± 13934	*p* = 0.8413	- IB vs IIA-
	Stage IIA - (n = 5)	32404 ± 28224		
	**% IL-17A in benign CD4 vs stage**		**Mann-Whitney test**	
	- Stage IB (n = 5)	1.10 ± 1.24	*p* = 0.4127	- IB vs IIA-
	Stage IIA - (n = 5)	2.57 ± 1.63		
**S2B**	**% CD69+CD103+ in benign CD4 vs stage**		**Mann-Whitney test**	
	- Stage IB (n = 5)	2.16 ± 1.60	*p* = 0.5368	- IB vs IIA-
	Stage IIA - (n = 5)	7.09 ± 12.57		
	**% CD69+CD103+ in benign CD8 vs stage**		**Mann-Whitney test**	
	- Stage IB (n = 5)	7.87 ± 8.49	*p* > 0.9999	- IB vs IIA-
	Stage IIA - (n = 5)	5.50 ± 4.06		
